# Tumor Development, Growth Characteristics and Spectrum of Genetic Aberrations in the TH-*MYCN* Mouse Model of Neuroblastoma

**DOI:** 10.1371/journal.pone.0051297

**Published:** 2012-12-17

**Authors:** Agnes Rasmuson, Lova Segerström, Maria Nethander, Jennie Finnman, Lotta H. M. Elfman, Niloufar Javanmardi, Staffan Nilsson, John Inge Johnsen, Tommy Martinsson, Per Kogner

**Affiliations:** 1 Childhood Cancer Research Unit, Department of Women's and Children's Health, Karolinska Institutet, Stockholm, Sweden; 2 Department of Clinical Genetics, Institute of Biomedicine, University of Gothenburg, Sahlgrenska University Hospital, Gothenburg, Sweden; 3 Genomics Core Facility, Gothenburg University, Gothenburg, Sweden; 4 Department of Mathematical Statistics, Chalmers University of Technology, Gothenburg, Sweden; Ohio State University Comprehensive Cancer Center, United States of America

## Abstract

**Background:**

The TH-*MYCN* transgenic neuroblastoma model, with targeted MYCN expression to the developing neural crest, has been used to study neuroblastoma development and evaluate novel targeted tumor therapies.

**Methods:**

We followed tumor development in 395 TH-*MYCN* (129X1/SvJ) mice (125 negative, 206 hemizygous and 64 homozygous mice) by abdominal palpations up to 40 weeks of age. DNA sequencing of *MYCN* in the original plasmid construct and mouse genomic DNA was done to verify the accuracy. Copy number analysis with Affymetrix® Mouse Diversity Genotyping Arrays was used to characterize acquired genetic aberrations.

**Results:**

DNA sequencing confirmed presence of human *MYCN* cDNA in genomic TH-*MYCN* DNA corresponding to the original plasmid construct. Tumor incidence and growth correlated significantly to transgene status with event-free survival for hemizygous mice at 50%, and 0% for homozygous mice. Hemizygous mice developed tumors at 5.6–19 weeks (median 9.1) and homozygous mice at 4.0–6.9 weeks (5.4). The mean treatment window, time from palpable tumor to sacrifice, for hemizygous and homozygous mice was 15 and 5.2 days, respectively. Hemizygous mice developing tumors as early as homozygous mice had a longer treatment window. Age at tumor development did not influence treatment window for hemizygous mice, whereas treatment window in homozygous mice decreased significantly with increasing age. Seven out of 10 analysed tumors had a flat DNA profile with neither segmental nor numerical chromosomal aberrations. Only three tumors from hemizygous mice showed acquired genetic features with one or more numerical aberrations. Of these, one event corresponded to gain on the mouse equivalent of human chromosome 17.

**Conclusion:**

Hemizygous and homozygous TH-*MYCN* mice have significantly different neuroblastoma incidence, tumor growth characteristics and treatment windows but overlap in age at tumor development making correct early genotyping essential to evaluate therapeutic interventions. Contrasting previous studies, our data show that TH-*MYCN* tumors have few genetic aberrations.

## Introduction

Neuroblastoma is an extracranial childhood tumor of the sympathetic nervous system [1]. It displays a very heterogeneous clinical behavior with some tumors spontaneously regressing, whereas others are incurable despite aggressive treatment. Therefore, research has focused on finding specific molecular markers predictive of tumor responsiveness. One of the earliest discovered genetic markers and still one of the strongest predictors of poor prognosis, is amplification of the *MYCN* gene [2]–[4]. The prevalence of *MYCN* amplification in neuroblastoma patients is 20–30% and the overall survival for these patients is 15–35% [1], [5].

To investigate if MYCN by itself can contribute to neuroblast transformation, Weiss *et al.* developed a transgenic mouse model with human MYCN expression targeted to migrating cells of the neural crest, by aid of the tyrosine hydroxylase promotor (TH-*MYCN*, [6]). The TH-*MYCN* mice develop neuroblastoma-like tumors, due to a deficient process of neural crest cell deletion during early life, demonstrating that high MYCN expression can initiate tumorigenesis [6], [7]. The penetrance and latency are dependent on the gene dosage. All homozygous mice have been reported to develop tumors within seven to eight weeks of age whereas 27–65% of the hemizygous mice develop tumors about 13 weeks of age [6], [8]–[10].

The TH-*MYCN* model has been appreciated for its resemblance to its human counterpart and is now the most widely used transgenic model in neuroblastoma research. The TH-*MYCN* tumors predominantly arise in a paraspinous location in the abdomen. Gross metastases are rare, whereas local spread to lymph nodes and microscopic metastases to the liver, kidney, lung, ovary, testes, brain, bone marrow and muscles more commonly are observed [6], [7], [10], [11]. Histologically, the tumors show the presence of specific neuronal markers, small, round, blue cells and a varying degree of neuronal differentiation [6], [11]. To provide a model of tumor progression, two tumor staging systems have been developed, one based on tumor weight and one on tumor size and regional spread [10], [12].

The human *MYCN* cDNA transgene is integrated on chromosome 18, and further *MYCN* transgene amplification has been observed [7], [13], [14]. Early PCR analysis of microsatellite markers and comparative genomic hybridization (CGH) analysis showed that tumors derived from this model carry many genetic aberrations, some similar to the human disease [13], [15].

In this study we have sequenced the *MYCN* transgene, and performed copy number analysis of hemizygous and homozygous tumors. In addition, we provide data on tumor incidence and growth dynamics, information that is essential for the therapeutic application of the TH-*MYCN* model of neuroblastoma.

## Materials and Methods

### Ethics Statement

This study was carried out in strict accordance with Swedish national regulations (SFS 1988:534), and with the recommendations in the Rules for working with laboratory animals of the Karolinska Institutet. The protocol was approved by the Committee on the Ethics of Animal Experiments of Northern Stockholm (Permit Number: 39/08).

### Mice

The TH-*MYCN* mouse colony was obtained from the Mouse Model of Human Cancer Consortium (MMHCC) repository as an N16 backcross to 129X1/SvJ. Mice were back crossed for two generations to wt 129X1/SvJ females purchased from Jackson Laboratories as specified by the MMHCC, and kept as a continuous inbred colony on the 129X1/SvJ background. Breeders were kept in harems and pups were biopsied at two weeks of age. The animals were housed at a max of six per cage in an enriched environment with access to food and water *ad libitum*. All animals underwent abdominal palpations two to three times weekly to monitor tumor development. After genotyping or at palpable tumor development some animals, depending on status of transgene, were randomized to other intervention. At that time the animal was censored concerning evaluation of spontaneous tumor development and excluded from treatment window, respectively. After tumor development, animals were monitored closely and euthanized using carbon dioxide, upon signs of discomfort and tumor-related problems according to the criteria of Karolinska Institutet, or when this could be anticipated due to the tumor burden and rapid tumor progression. All efforts were made to minimize suffering. The treatment window was defined as the number of days from the detection of a palpable tumor to the day of sacrifice or death, due to tumor-related signs of discomfort or heavy tumor burden.

### Genotyping

DNA was extracted from ear clips and tail biopsies using DNeasy Blood and Tissue kit (Qiagen, Sollentuna, Sweden). The PCR was performed in a total reaction volume of 20 µL containing 0.2 µM of each primer, 2% DMSO, 60 ng of DNA and HotStarTaq® Plus master mix (Qiagen). Samples were heated for 5 min at 95°C and amplified for 34 cycles of 30 s at 96°C, 30 s at 60°C, and 40 s at 72°C, and finally at 72°C for 10 min. The primers used were as follows: Chr18F1: 5′-ACTAATTCTCCTCTCTCTGCCAGTATTTGC-3′, Chr18R2: 5′-TGCCTTATCCAAAATATAAATGCCCAGCAG-3′, and OUT1: 5′-TTGGCACACACAAATGTATATACACAATGG-3′ (Applied Biosystems, Boston, MA, USA, [14]). PCR products were separated by 1.5% agarose gel electrophoresis, stained with GelRed (Biotium) or ethidium bromide and photographed under UV-light.

### Sequencing

The pTH-*MYCN* plasmid was a kind gift from Prof. Weiss at University of California San Francisco. For sequencing, 200 ng of plasmid DNA, 5 pmol of each primer and 1 µL of BigDye® Terminator v.3.1 (Applied Biosystems) were used for each reaction. Samples were heated for 1 min at 96°C followed by 1 min at 98°C, and run for 36 cycles of 10 s at 98°C, 5 s at 50°C, and 4 min at 60°C. The following primers were used to sequence human *MYCN* cDNA, forward primers; F1: 5′-GAAAGAAGCCCTCAGTCGC-3′, F2: 5′-TTTCCCGTGAACAAGCGCG-3′, and F3: 5′-TTTCTCACGCTCAGGGACC-3′, and reverse primers; R1: 5′-ATCAAAATGTGCAAAGTGGC-3′, R2: 5′-TGACCACGTCGATTTCTTCC-3′, and R3: 5′-AGCTGTGCTCCGCGAAGCCAC-3′ were used (Thermo Fisher Scientific, Ulm, Germany). Samples were sequenced in an ABI 3730 DNA Analyzer (Applied Biosystems) and analyzed using Sequencing Analysis 5.3.1 (Applied Biosystems). The resulting six sequences were aligned using SeqMan (DNASTAR, Madison, WI, USA). For sequencing mouse genomic DNA, DNA was isolated as described above. The *MYCN* insert was amplified using the forward primer 5′nmyc 1 and reverse primer 3′nmyc 1, as outlined above. Amplification was performed in a 20 µL total volume of reaction, containing 2.5 units of HotStarTaq (Qiagen), 40 ng DNA, 0.5 µM of each primer and Q solution (Qiagen). Samples were heated for 15 min at 95°C, and amplified in 36 cycles of 1 min at 94°C, 1 min at 58.9°C, 3 min at 72°C, followed by 10 min at 72°C. PCR products were separated by 1.5% agarose gel electrophoresis and photographed under UV light. All unused nucleotides and single stranded DNA was degraded using ExoSAP-IT (GE Healthcare, Uppsala, Sweden). The product was sequenced with bidirectional sequencing using the same primers and conditions as described above albeit only one primer at the time. The contig sequence has been deposited in Genebank, accession number BankIt1571050 Seq JX952197.

### High Frequency Ultrasound

The Visual Sonic Vevo® 2100 system was used for ultrasound imaging. Mice were anaesthetized with 1.5 to 2.5% isoflurane in O_2_ at 1 liter/min. Mice were maintained under continuous isoflurane anesthesia and placed on the heated Vevo mouse handling table which was mounted on the Vevo 2100 imaging station. Mouse fur was removed with a commercially available depilatory cream. Prewarmed ultrasound coupling gel (Aquasonic 100; Parker Laboratories, Inc, Fairfield, NJ) was applied directly to the skin. Mice were then scanned from the ventral body wall using either the MS250 or the MS55D real-time MicroScan™ transducer and the Vevo 2100 imaging system.

### Copy number analyses of TH-*MYCN* mouse tumor array data

DNA, from 10 tumors derived from 10 different mice and normal liver were subjected to array analysis using the Affymetrix Mouse Diversity Genotyping Array service. TG1-TG5 were derived from mice developing tumors early and TG6-TG10 from mice developing tumors late. TG1-TG4 showed by genotyping to be derived from homozygous mice, and TG5-TG10 from hemizygous mice. Genotyping data and data from CEL files were analyzed as described here briefly. We used array data generated from eleven “test DNA samples”; one from a healthy liver of a negative animal and 10 from tumors from 10 different TH-*MYCN* animals. The probe level intensities were extracted from the CEL-files using Affymetrix Power Tools (www.affymetrix.com). The data processing was done in R and only perfect match Genotype probes were used. For each sample and marker a *marker intensity* was constructed from the median of the corresponding probe intensities. A *normalized intensity* was then formed for each marker of the 10 test DNA samples by calculating the ratio between the test samples marker intensity and the corresponding intensity for the healthy liver of the syngenic animal. Finally the normalized intensities are plotted against the marker positions.

### Statistical analysis

A chi-square goodness of fit test was used to test the Mendelian proportions (25%, 50%, 25%) of the pups born from TH-*MYCN* breeding. The two-sided unpaired t-test was performed on log-transformed data to compare time to tumor development and the treatment window for hemizygous versus homozygous mice, respectively. Fisher's exact test was used to evaluate the spontaneous deaths. Linear regression analysis and two-sided Spearman test were used to test the correlation between age at palpable tumor and the treatment window. GraphPad was used for all statistical analyses (GraphPad software Inc., San Diego, CA, USA).

## Results

### Sequencing of the *MYCN* transgene

No previous study has confirmed that the *MYCN* transgene integrated into the TH-*MYCN* mouse genomic DNA corresponds to human *MYCN* cDNA. Therefore we decided to sequence the *MYCN* insert cloned into the original plasmid, pTH-*MYCN*, and genomic TH-*MYCN* mouse DNA. An assembly of the six resulting sequences that were obtained from sequencing pTH-*MYCN* resulted in one contig sequence covering a total of 2872 nucleotides. The 609–2179 nucleotides were a perfect match of human *MYCN* cDNA (Figure S1).The contig sequence obtained from sequencing genomic TH-*MYCN* mouse DNA was identical to the plasmid sequence (data not shown).

### TH-*MYCN* breeding

We used primers published by Haraguchi and Nakagawara to genotype a total of 395 mice from 80 litters [14]. Negative and hemizygous mice were used for breeding. Negative x hemizygous and hemizygous x hemizygous breeding resulted in a mean litter size of 5.4 and 4.8, respectively. The negative x hemizygous breeding resulted in 20 litters with a total of 108 live off-springs where of 44 negative (41%) and 64 hemizygous (59%) mice (p = 0.054). The hemizygous x hemizygous breeding resulted in 60 litters with a total of 287 live off-springs where of 81 negative (28%), 142 hemizygous (50%) and 64 homozygous (22%) mice (p = 0.36).

### Tumor free survival and time to tumor development

**Figure 1:**
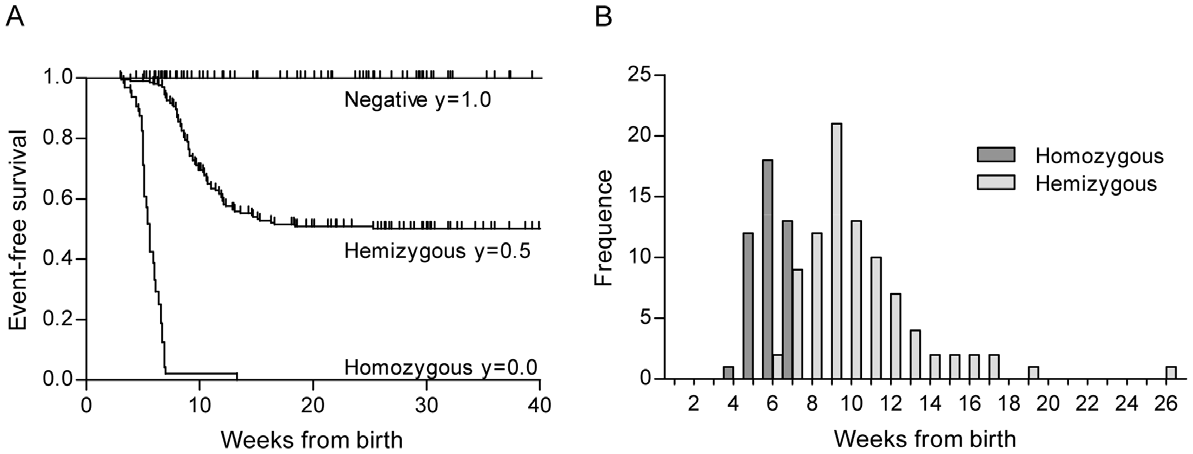
Tumor development of TH-*MYCN* mice on the 129X1/SvJ background. **A.** Event-free survival. The y-axis shows the proportion of animals that remain free of an event, here defined as palpable tumor development, spontaneous death by unknown cause including missing animals, and euthanization due to general signs of discomfort. The x-axis shows weeks from birth. Negative n = 125, hemizygous n = 206 and homozygous n = 64. **B.** Frequency distribution diagram showing the age at palpable tumor. Homozygous mice developed palpable tumors between 4.0–6.9 weeks of age, mean of 5.6 and median of 5.4 weeks (n = 45). The hemizygous mice that developed tumors were palpated with a tumor between 5.6–19 weeks of age, mean 9.9 and median 9.1 weeks (n = 88). The two-sided unpaired t test was used for comparison between the two groups p<0.01.

Two hundred and six hemizygous mice were followed with repeated abdominal palpations up to 40 weeks of age during which 88 developed a tumor within 5.6–19 weeks of age. Only one mouse developed its tumor later, at 26 weeks of age. Five hemizygous mice spontaneously died, without any palpable tumor, at the age of 3.1–12 weeks. Three hemizygous mice, aged 8.7–12 weeks, had to be euthanized due to general signs of discomfort before any tumor was detected. Kaplan-Meier analysis showed that the event-free survival probability for hemizygous mice was 50% (Figure 1A). An event was defined as palpable tumor development, spontaneous death by unknown cause which includes missing animals, or euthanizing due to general signs of discomfort. For the hemizygous mice that developed tumors the mean and median age at palpable tumor was 9.9 and 9.1 weeks, respectively (Figure 1B). Sixty-four homozygous animals were followed up to 13 weeks of age. During this time, 45 developed a palpable tumor within 4.0–6.9 weeks of age, median 5.4 and mean 5.6 weeks. Nine homozygous animals died spontaneously without a palpable tumor, aged 3.3–13 weeks, and one mouse had to be euthanized at 3.4 weeks of age due to general signs of discomfort before any tumor was detected (Figure 1A and B). According to the Kaplan-Meier analysis, the event-free survival probability for homozygous mice was 0%. Hemizygous mice developed palpable tumors significantly later than homozygous mice (p<0.01) but, as shown in Figure 1B, the two groups overlap. No differences in tumor development were found between female and male mice (data not shown). No animals negative for the *MYCN* transgene spontaneously died or had to be preterm euthanized during the time of follow up, significantly less compared to animals with one or two *MYCN* transgenes (p<0.01). The homozygous mice were significantly more prone to spontaneous deaths compared to hemizygous mice (p<0.01).

### Tumor imaging using high resolution ultrasound

**Figure 2:**
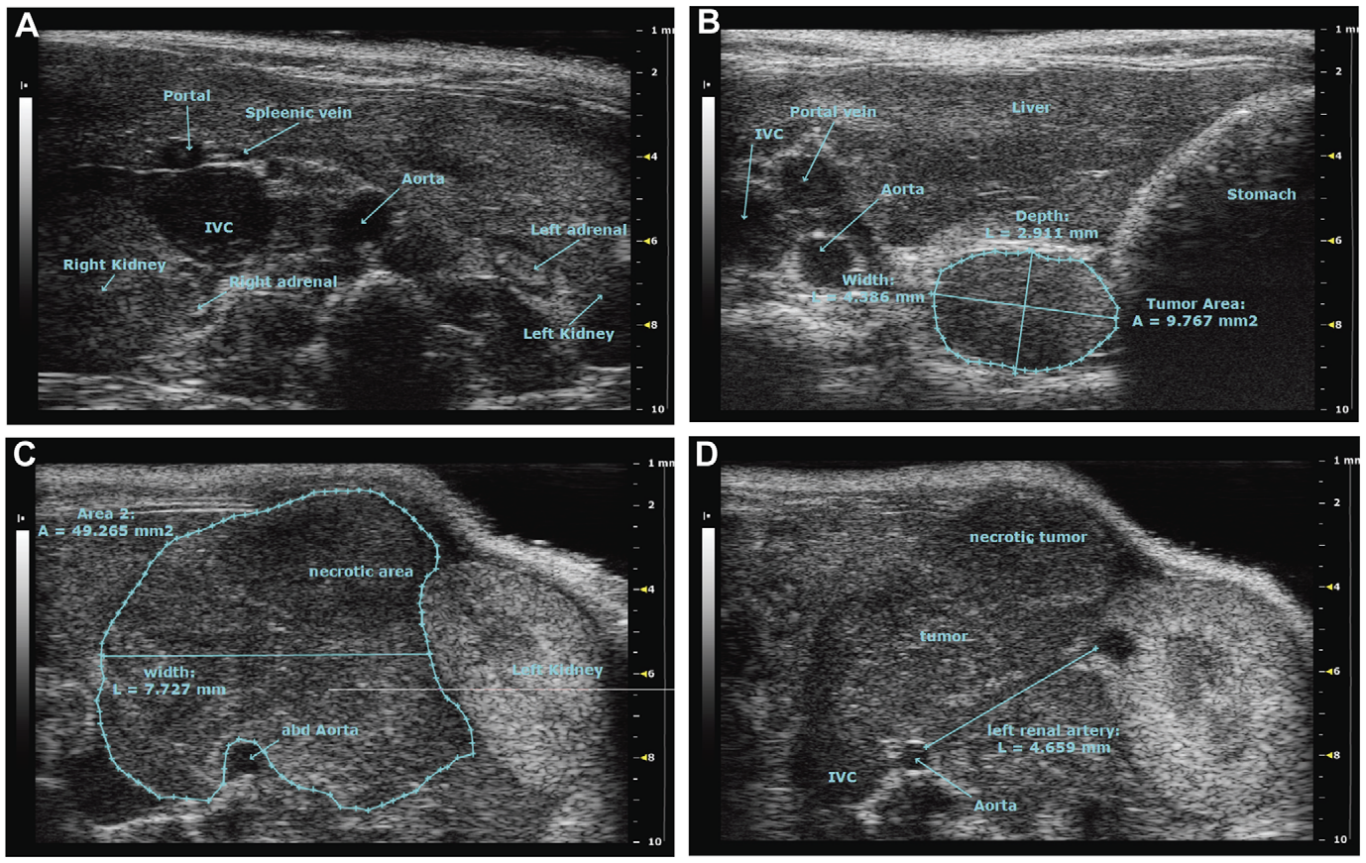
Tumor imaging using high resolution ultrasound. **A**. Tumor free animal visualizing abdominal landmarks shown by high resolution ultrasound. **B**. Detection of a non-palpable small tumor lesion (9.8 mm^2^) growing on the left side of the spine attached to the muscles. **C** and **D**. Imaging of a large palpable tumor (49 mm^2^). Growing around the aorta and inferior vena cava (IVC), displacing the left kidney. The pictures shown are transverse sections, oriented with the dorsal side down and the ventral up.

The TH-*MYCN* mice predominantly develop abdominal tumors which can be palpated when sufficiently large. Therefore we evaluated high frequency ultrasound for tumor imaging. As shown in Figure 2B, the tumors arise in a paraspinous location in the abdomen. Upon further growth these tumors displace vital blood vessels and attach to the kidneys, adrenals, liver and intestine (Figure 2C and D).

### Differences in treatment window between homozygous and hemizygous mice

**Figure 3:**
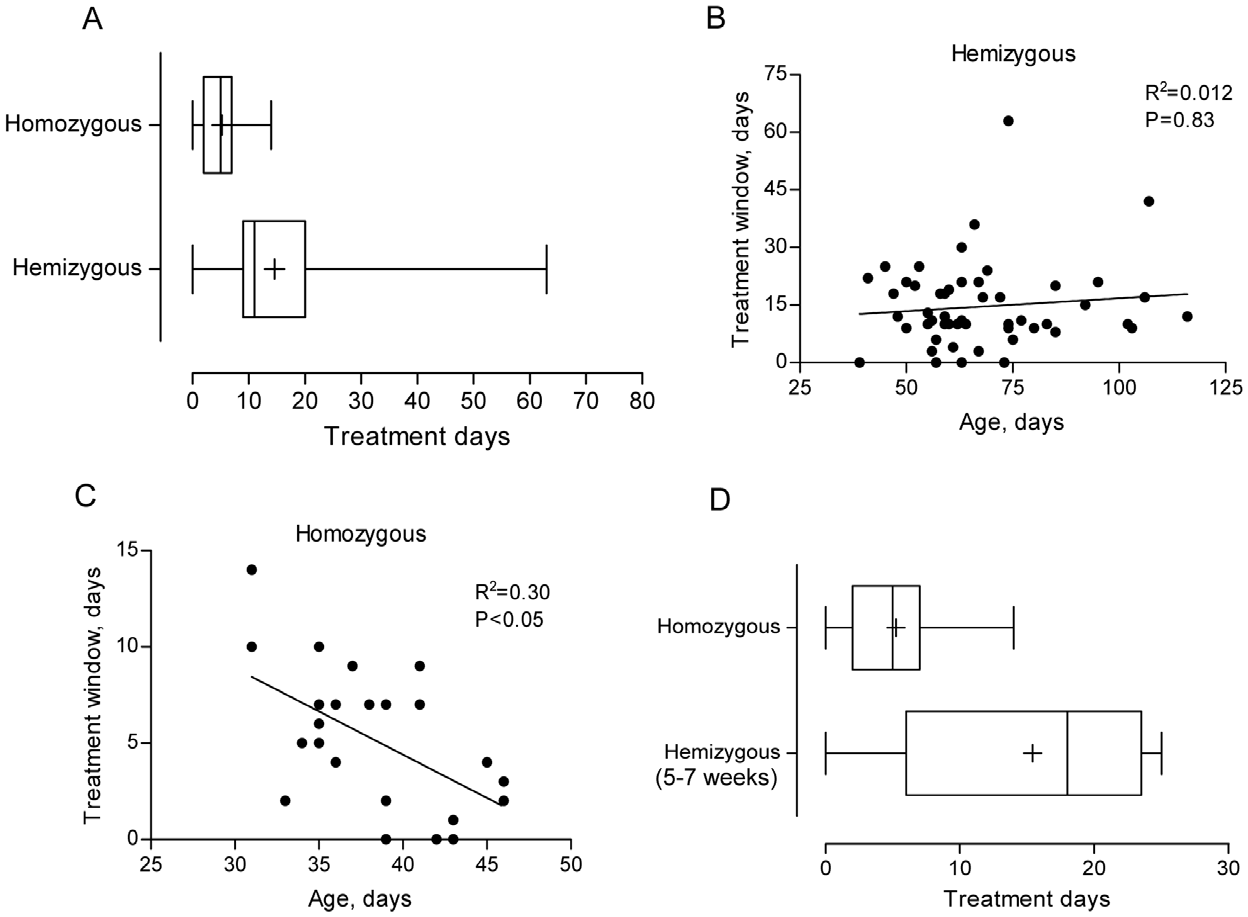
Time from a palpable tumor to sacrifice – the treatment window. **A.** Treatment window for homozygous and hemizygous mice. Mean number of treatment days for homozygous mice was 5.2 days (median 5, n = 30) and for hemizygous mice 15 days (median 11, n = 54). Statistical analysis was performed using two-sided *t* test on log-transformed data; p<0.01. **B.** Treatment window for hemizygous mice as a function of age at tumor palpation. Linear regression analysis yielded R^2^ =  0.012 and the correlation was assessed using two-sided Spearman test; p = 0.83. **C.** Treatment window for homozygous mice as a function of age at tumor palpation. Linear regression analysis yielded R^2^ =  0.30 and the correlation was assessed using two-sided Spearman test; p<0.05. **D.** Treatment window for homozygous mice versus hemizygous mice with an early tumor development (5.6–7.0 weeks of age). Mean number of treatment days for homozygous mice was 5.2 days (median 5, n = 30) and for hemizygous mice with an early tumor development 15 days (median 18, n = 5). Statistical analysis was performed using two-sided t test on log-transformed data; p<0.01. Box plots show median, inter quartile range (25–75%), min and max, and + represent the mean.

The time from a palpable tumor to sacrifice, i.e. the treatment window, for hemizygous mice ranged from 0–63 days, mean 15 and median 11 days (n = 54). Homozygous mice had a significantly shorter treatment window ranging from 0–14 days, mean 5.2 and median 5 days (n = 30, p<0.01, Figure 3A). There was no correlation between age at palpable tumor and the treatment window for hemizygous mice (Figure 3B). In contrast, for homozygous there was a significant correlation between age at tumor development and the treatment window, with a decreasing treatment window upon increasing age at tumor development (p<0.05, Figure 3C). Importantly, hemizygous mice with an early palpable tumor had a significantly longer treatment window, mean 15 and median 18 days, compared to homozygous mice developing tumor at the same age, mean 5.2 and median median 5 days, (p<0.01, Figure 3D).

### Analyses of copy number changes in TH-*MYCN* tumors

**Figure 4:**
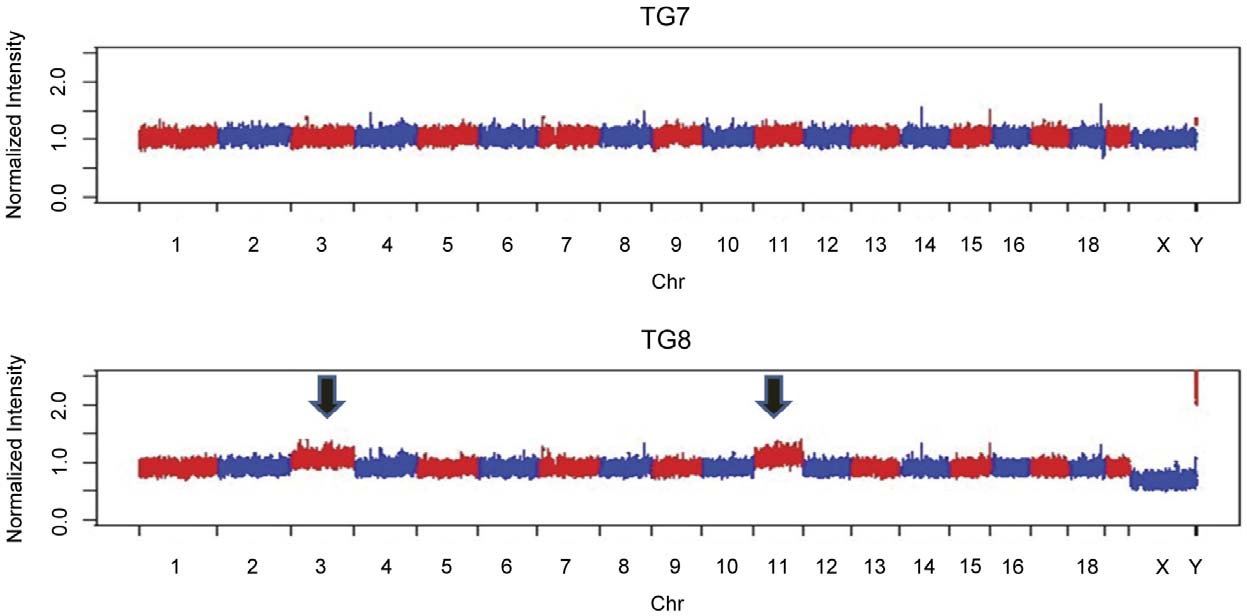
Whole genomic profile of two representative cases of the ten analyzed TH-*MYCN* tumors. TG1–TG7 showed flat profiles with no segmental or numerical aberrations (TG7 is shown here). TG8, TG9 and TG10 showed a few numerical aberrations. TG8 shown here display whole chromosome gain of mouse chromosome 3 and 11 (indicated by arrows).

Of the ten tumor samples analyzed for copy number variations seven tumors (TG1–TG7), including all tumors from homozygous mice, had flat profiles, i.e. with neither segmental nor numerical aberrations (Table 1, Figure 4). Three samples (TG8–TG10), all derived from hemizygous mice, had one or two numerical changes (Table 1). All three tumor samples had whole chromosome gain of mouse chromosome 3. In addition TG8 had whole chromosome gain of MMU11 (Figure 4) while TG9 had whole chromosome loss of MMU10 and 11. No segmental aberrations were detected in any of the ten tumors. MMU3 that was gained in TG8, TG9 and TG10, is syntenic to the following regions in the human genome (from top-down): Chr1:68,589,539-158,154,741; Chr13:34,480,325-41,248,741; Chr3:149,055,024-182,817,955; Chr 4:95,284,699-141,191,469; Chr4:150,966,383-163,096,512; Chr8: 64,073,505-67,358,470; Chr8; 76,205,056-87,056,724. MMU10 that was lost in TG9 is syntenic to the following regions in the human genome: Chr10:55,366,623-74,859,261; Chr12:55,349,827-108,177,689; Chr19:281,181-2,497,327; Chr19:15,048,169-15,296,036; Chr2:109,065,537-110,379,204; Chr21:45,138,911-48,084,912; Chr22:24,092,823-24,633,370; Chr22:32,783,299-33,472,414; Chr6:100,534,800-154,998,070. MMU11 that was gained in TG8 is syntenic to the following regions in the human genome: Chr1:227,919,753-228,704,432; Chr17:2-20,222,700; Chr17:25,556,525-81,153,936; Chr2:53,887,805-68,694,726; Chr22:29,167,187-32,022,116; Chr5:130,483,100-173,711,408; Chr5:177,530,538-180,682,008; Chr7:43,906,144-55,317,931. Thus, genomic regions corresponding to almost all of human chromosome 17 were gained in one tumor, TG8. No event corresponding to a human 1p-deletion or a human 11q deletion commonly found in human neuroblastoma [16] could be detected in any of the ten tumors. The SNP data were also scored (data not shown but available on request) and we could show that in a typical experiment with DNA from normal tissue of a negative 129X1/SvJ mouse we retrieved 579,576 calls of which 571,577 (98.6%) were homozygous for either allele while 7999 (1.4%) were heterozygous.

**Table 1:** Summary of genomic aberrations in ten different TH-*MYCN* tumors.

		Mouse chromosomes																				
Tumor no.	Genotype	1	2	3	4	5	6	7	8	9	10	11	12	13	14	15	16	17	18	19	20	X
TG1	+/+^a^																					XX
TG2	+/+																					X
TG3	+/+																					XX
TG4	+/+																					XX
TG5	+/−b																					XX
TG6	+/−																					X
TG7	+/−																					XX
TG8	+/−			wcg^c^								wcg										X
TG9	+/−			wcg							wcl^d^											XX
TG10	+/−			wcg																		X

Abbreviations:

a homozygous;

b hemizygous;

c whole chromosome gain;

d whole chromosome loss.

## Discussion

The MYCN oncogene plays an important role for neuroblastoma development, tumor aggressiveness and clinical prognosis [1]. The appropriate use of a neuroblastoma specific genetic engineered mouse model has shown great promise for the development of novel therapies [8]. We have in this work further characterized the TH-*MYCN* model and detected specific differences in tumor incidence, time to tumor development and therapeutic window between mice hemizygous and homozygous for the TH-*MYCN* transgene, supporting the role of early and correct genotyping for future evaluation of targeted therapeutic interventions. Tumors from transgenic mice show very few acquired genetic aberrations as investigated with a mouse specific single nucleotide polymorphism (SNP) array.

The *MYCN* cDNA clone used to create the TH-*MYCN* transgenic mouse model was first published by Ramsay and co-workers in 1986 [17]. To confirm that the insert is intact and corresponds to human *MYCN* cDNA we sequenced the *MYCN* insert in the original plasmid that was used to create the TH-*MYCN* model and genomic TH-*MYCN* mouse DNA. The resulting contig sequences revealed that the sequences are an exact match of the entire amino acid coding part, located in exon 2 and 3 of human *MYCN* cDNA (Figure S1).

Genotyping a total of 287 animals from hemizygous x hemizygous breeding showed that the genotype distribution of the pups surviving two weeks of age, was as would be expected (p = 0.36). This result thus demonstrates that carrying one or two copies of the *MYCN* transgene does not give any obvious developmental disadvantages in prenatal or early life.

The TH-*MYCN* tumor penetrance is dependent on the mouse strain, with some being more susceptible as the 129X1/SvJ, and others more resistant [6]. This variability may be due to the differential expression of yet unidentified strain-specific modifiers. However, differences in tumor penetrance has also been shown for different breeding facilities [18]. This could, in part, be due to the so-called founder effect where the genetic diversability of a population is constrained to that of the original founders and consecutive genetic drift and mutations upon breeding. As shown in Figure 1A, the event-free survival probability in this study was 50% for hemizygous mice and 0% for homozygous mice, respectively. One homozygous mouse that spontaneously died at 13 weeks of age, stands out in the Kaplan-Meier analysis. By an unknown cause, the behavior of the tumor of this homozygous mouse better fits into the hemizygous group of animals. No negative animals died spontaneously or had to be euthanized due to general signs of discomfort during the follow up. This indicates that these events among hemizygous (4%) and homozygous (19%) mice are related to tumor development. Other studies have shown that the TH-*MYCN* tumors can be multifocal and tumors may also arise in thoracic paraspinal ganglia [6], [10], which have also been rarely observed in this colony. Therefore we decided to include spontaneous deaths and euthanizing due to general signs of discomfort as an event in the Kaplan-Meier analysis.

Homozygous mice developed palpable tumors significantly earlier than hemizygous mice (p<0.01). The median age at palpable tumor for homozygous mice was 5.4 weeks and 9.1 weeks for hemizygous mice, respectively (Figure 1B). However, some hemizygous mice developed palpable tumors as early as homozygous mice (Figure 1B). Similarly, Teitz et al routinely detected small tumors in six to nine weeks old hemizygous mice, by ultrasound or magnetic resonance imaging [11]. Therefore, time to a palpable tumor is not suitable as a surrogate marker for determining genotype in this model when breeding on hemizygous x hemizygous mice.

Human neuroblastoma tumors can arise anywhere along the sympathetic axis but they most frequently arise in the abdomen, in the adrenal gland medulla [1], [19]. As visualized by the ultrasound images in Figure 2B–D, and which has previously been shown [11], the TH-*MYCN* mice mainly develop tumors in paraspinal ganglia. When the tumors grow larger they attach to and displace the adrenal glands, the kidneys, the aorta and other vital organs. Some tumors may also develop in a location more closely to the adrenal and kidney [11] and some tumors, that are difficult to visualize by ultrasound, arise in the thorax.

Many therapeutic studies using this model have started the treatment at the time of a palpable tumor [20]–[22]. We have followed 54 hemizygous, and 30 homozygous animals, from the day of a palpable tumor to sacrifice, here defined as the treatment window. Our results reveal that the treatment window is significantly different for hemizygous and homozygous mice, respectively. Hemizygous mice had a mean of 15 treatment days whereas homozygous mice only have 5.2 days (Figure 3A). This discrepancy could partly be explained by the difference in gene dosage, with a higher expression of MYCN driving a more rapid tumor growth. Another possibly important factor influencing the treatment window is the body size, where the homozygous mice are much younger and smaller at the time of tumor development. We also investigated the correlation between age at palpable tumor development, and the treatment window for hemizygous and homozygous mice, respectively (Figure 3B and C). The data show two different patterns depending on if the animal has one or two *MYCN* transgene copies. For the hemizygous mice, there is no correlation between age at palpable tumor and the treatment window (R^2^ = 0.012, p = 0.83). The rate of tumor growth is similar regardless of age at palpable tumor development. The treatment window is dependent on several factors including growth characteristics, location, body size and palpations skills. Nevertheless, for homozygous mice there is a significant correlation between age at palpable tumor and the treatment window (R^2^ = 0.30, p<0.05, Figure 3C). In addition, compared to homozygous mice, hemizygous mice with an equally early tumor development had a significantly longer treatment window, 5.2 versus 15 days (Figure 3D). These data suggests that the homozygous tumors are driven by a high MYCN expression whereas the hemizygous tumors are driven by other contributing mechanisms. Although there was a tendency (p = 0.16) that only tumors from hemizygous mice contained acquired genetic aberrations, we could not detect any significant difference between hemizygous and homozygous that could explain the different growth patterns (Table 1, Figure 4). Together, our data leads to a recommendation that therapeutic interventions must be based on proper genotyping. Treatment in homozygous mice can be started at a fixed age whereas treatment in hemizygous mice should be started at detection of a palpable tumor or a tumor visualized by ultrasound.

We used the Affymetrix® Mouse Diversity Genotyping Array for the analyses of copy number changes for ten tumor samples. The array interrogates approximately 600,000 SNP positions and 900,000 non-polymorphic regions. The array type used was chosen for these experiments on grounds of its very high probe density, and the non-polymorphic probes were used for the copy-number analyses. The SNP data were also scored and we could show that in DNA from normal tissue from a typical experiment we retrieved 579,576 calls of which 571,577 (98.6%) were homozygous for either allele while 7999 (1.4%) were heterozygous. The heterozygous positions were scattered over all chromosomes. It is likely that the very specific Affymetrix SNP array analysis profile generated here can serve as a quality control to confirm strain background in future experiments. The array system together with our in-house software enabled detailed, rapid and robust detection of gains and losses for all chromosomes. Only three out of ten tumor samples displayed aberrations, in total 5 changes (Table 1, Figure 4). All these changes appeared only in tumors from hemizygous mice and were numerical whole-chromosome changes, four gains and one loss. As earlier also shown by Hackett *et al*. the most common aberration was gain of mouse chromosome 3 [13]. All three cases with aberrations showed this. Mouse chromosome 3 is syntenic to parts of the following human chromosomes: 1, 3, 4, 8, and 13. An additional gain was detected on mouse chromosome 11. This was also detected as the second most common gain in the set of Hackett *et al.* [13]. This chromosome contain regions that are syntenic to human chromosome 17, a chromosome that is gained either as a segmental or as a numerical aberration in most human neuroblastoma tumors [1], [5], [16]. No segmental changes were detected in the set of tumors analyzed by us. In contrast to the study by Hackett *et al*., the majority of tumors displayed a flat profile (7 of 10; 70% flat profile), i.e. no aberrations, neither segmental nor numerical (Figure 4, [13]). In the set of Hackett *et al*. from 2003 only 5% of tumors showed a flat profile [13]. It should however be noted that Hackett et al. used a mixed F1 strain background for their study (i.e. cross of transgenic C57BL/6J to wild-type Mus musculus castaneus mice) with the intention to obtain as much chromosomal aberrations as possible [13], [15]. Hackett et al. also observed a much longer time to tumor development and a lower tumor incidence in this setting.

In conclusion, our data show that the tumor latency and growth is dissimilar in hemizygous and homozygous TH-*MYCN* mice, and indicates that the tumor growth is driven by different mechanisms in the two genotypes. This emphasizes the importance of genotyping in evaluating therapeutic interventions, since hemizygous and homozygous mice overlap in age at tumor development and have different treatment windows.

## Supporting Information

Figure S1 **Sequencing of the** ***MYCN*** **transgene.** The human *MYCN* cDNA nucleotide sequence present in the in the plasmid that was used to create the TH-*MYCN* model. The underlined nucleotides match human *MYCN* cDNA (only sequence from 659 to 2250 is shown).
(TIF)
