# Supplementary figures and images for: Tumor Development, Growth Characteristics and Spectrum of Genetic Aberrations in the TH-MYCN Mouse Model of Neuroblastoma
Source: PLoS One. 2012 Dec 17;7(12):e51297. doi: 10.1371/journal.pone.0051297 (PMC3524187; doi:10.1371/journal.pone.0051297)

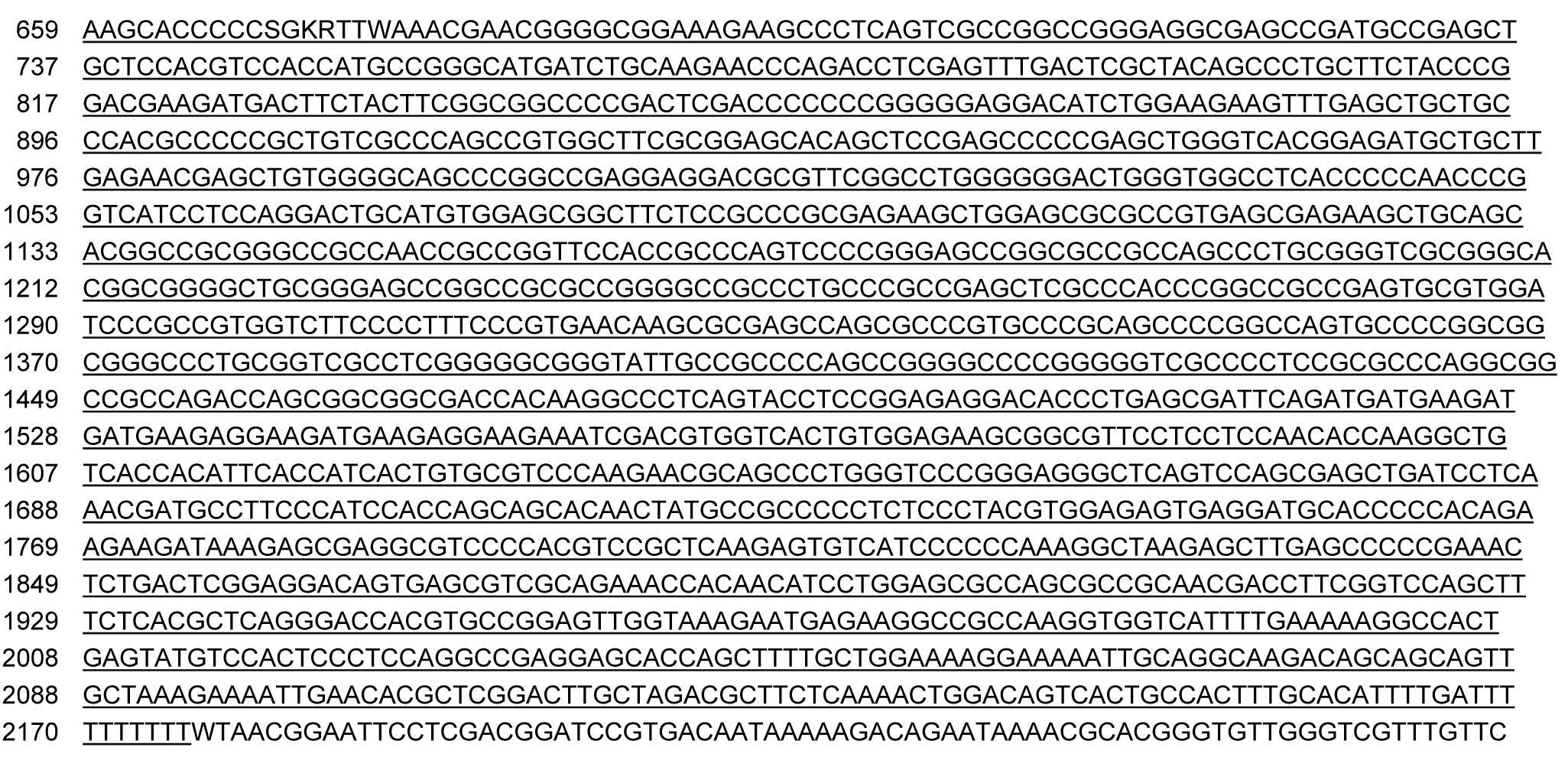

Supplement: Figure S1 — Sequencing of the MYCN transgene. The human MYCN cDNA nucleotide sequence present in the in the plasmid that was used to create the TH-MYCN model. The underlined nucleotides match human MYCN cDNA (only sequence from 659 to 2250 is shown). (TIF) [file pone.0051297.s001.tif]
